# Variation of honey bee wings in southwestern Asia

**DOI:** 10.1038/s41597-025-06234-8

**Published:** 2025-12-10

**Authors:** Julita Machlowska, Irfan Kandemir, Ayça Özkan Koca, Vakhtang Kakhniashvili, Yehya Alattal, Ahmad Alghamdi, Adam Tofilski

**Affiliations:** 1 University of Agriculture in Krakow, Krakow, Poland; 2 Ankara University, Ankara, Türkiye; 3 Maltepe University, İstanbul, Türkiye; 4 Georgian Beekeepers Union, Tbilisi, Georgia; 5 King Saud University, Riyadh, Saudi Arabia

**Keywords:** Entomology, Biogeography

## Abstract

The native range of the honey bee (*Apis mellifera*) includes Europe, Africa, and southwestern Asia. It is widely recognized that the genetic diversity of this species is being eroded in some regions due to the commercial supply of queen bees and migratory beekeeping. Other stressors present in some regions include agrochemicals, new parasites, and pathogens. Therefore, it is crucial to monitor the biodiversity of honey bees. A large dataset comprising 17,457 honey bee wings from 1,557 colonies in nine southwestern Asian countries was analyzed using 19 morphometric landmarks identified on forewing images. The results show that there is significant variation in wing shape across southwestern Asia. Pairwise comparisons revealed significant differences between most geographic regions. Wing geometric morphometrics is a cost-effective method that does not require sophisticated equipment and could be used by both scientists and beekeepers. The collection of wing images presented here, accessible through the Zenodo website, may facilitate the description and conservation of honey bee biodiversity in southwestern Asia.

## Background & Summary

The honey bee (*Apis mellifera* Linnaeus 1758) exhibits remarkable variability in its natural range, comprising at least 33 distinct subspecies (geographic races)^1–4^. It is native to Europe, Africa, and southwestern Asia, where it has adapted to diverse bioclimatic conditions^5^. Four major evolutionary lineages are recognized within the species: the African lineage (A), the Central and Eastern European lineage (C), the Western and Northern European lineage (M), and the Middle Eastern and Central Asian lineage (O)^3,6^. In recent years, honey bees from Ethiopia, originally designated as lineage A, have been reclassified as lineage Y, and another lineage, Z, has been designated in Syria and Lebanon^7,8^. The natural distribution of the honey bees has been altered by human activities, resulting in the expansion of their range and the replacement of some local populations with breeding lines preferred by beekeepers^9–11^. Commercial trade, long-distance migratory beekeeping and large-scale queen rearing can lead to a loss of genetic variability and consequently to a loss of local adaptations^9^. In some regions, other stressors are also present, including the use of agrochemicals, new parasites and pathogens, and climate change^12^. It is therefore of the greatest importance to conserve the underlying genotypic variation to ensure the continued survival of honey bees in their natural distribution^13^.

In southwestern Asia, there is the eastern limit of the native distribution of honey bees^14^. Deserts and high mountain ridges formed barriers to the natural dispersal of this species^3^. In this region, there is mainly lineage O^4^. Within this lineage Ruttner^3^ recognized seven well-defined subspecies: *Apis mellifera adami* Ruttner 1975, *Apis mellifera anatoliaca* Maa 1953, *Apis mellifera caucasia* Pollmann 1889, *Apis mellifera cypria* Pollmann, 1879, *Apis mellifera meda* Skorikov 1929, *Apis mellifera remipes* Gerstäcker 1862, and *Apis mellifera syriaca* Skorikov 1929. *Apis mellifera adami* and *A. m. cypria* are endemic subspecies of the islands of Crete and Cyprus, respectively. *Apis mellifera anatoliaca* occurs in most of Turkey^15^ and is bordered by *A. m. caucasia* to the northeast^16^, *A. m. remipes* to the east, and *A. m. meda* to the southeast^3^. *Apis mellifera syriaca* occurs on the eastern coast of the Mediterranean Sea. A new subspecies of *Apis mellifera pomonella* Sheppard & Meixner 2003, belonging to lineage O, was discovered 20 years ago in the Tien Shan Mountains of Kazakhstan^14^. Another subspecies, *Apis mellifera sinisxinyuan* Chen *et al*. 2016, belonging to lineage M, was described further south in China^1^. In addition, *Apis mellifera jemenitica* Ruttner 1976, belonging to lineage A, occurs in the Arabian Peninsula^3^. Beekeeping practices affected the native populations to some degree^17^. Introgression can differ between regions, and information about this problem is limited. Introgression may be more prevalent in the Levant region^18–21^ than in the Arabian Peninsula^22^.

The aim of this study is to establish a comprehensive online database of honey bee wing images. The images were newly collected or obtained from a number of previous studies, with the intention of providing a reference source showing the diversity of these bees in southwestern Asia. This approach will allow comparison of geographic regions and facilitate the detection of changes over time due to hybridization between native and non-native bees^23^. The wing images have been annotated with coordinates for 19 landmarks that can be used for future comparisons. Additionally, we provide an R Markdown document^24^ that analyzes the landmark coordinates.

## Methods

### Material and landmark digitization

**Fig. 1:**
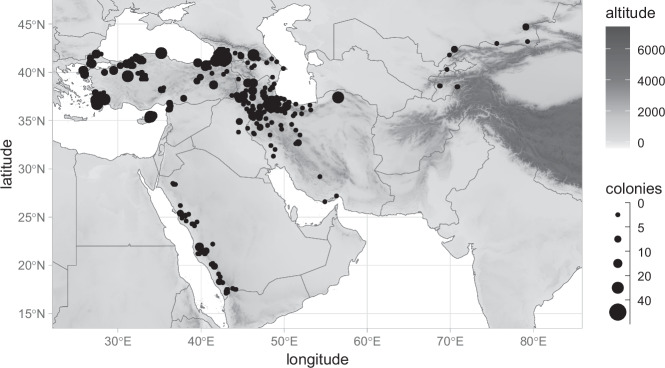
The map shows the study area. Black dots indicate locations from which samples were collected. The size of the dots corresponds to the number of colonies per location.

The present study used 17,457 forewing images of honey bee workers (non-reproductive females) from 1,557 colonies in nine countries in southwestern Asia (Fig. 1). The countries included Azerbaijan (114 wings from 14 colonies), Cyprus (654 wings from 73 colonies), Georgia (3,579 wings from 102 colonies), Iran (3,399 wings from 401 colonies), Iraq (91 wings from 11 colonies), Kazakhstan (442 wings from 29 colonies), Saudi Arabia (1,397 wings from 127 colonies), Tajikistan (88 wings from 5 colonies), and Turkey (7,693 wings from 795 colonies). The number of workers per colony ranged from four to 40, with an average of 11.2. Data for Kazakhstan are already publicly available^25,26^. We only used colonies from Kazakhstan that did not originate from honey bee queen breeders and were considered native. Some of the data from Azerbaijan^27^, Cyprus^28^, Iran^29^, Iraq^30^, Saudi Arabia^31–33^, Turkey^15,30,34–36^ have been previously presented in a different context. Data from Georgia and Tajikistan are presented for the first time. A considerable part of southwestern Asia is unsuitable for honey bees due to the presence of deserts or high mountains. The data presented here cover most of the area suitable for honey bee survival; however, there are no data from certain countries such as Syria and Jordan.

**Fig. 2:**
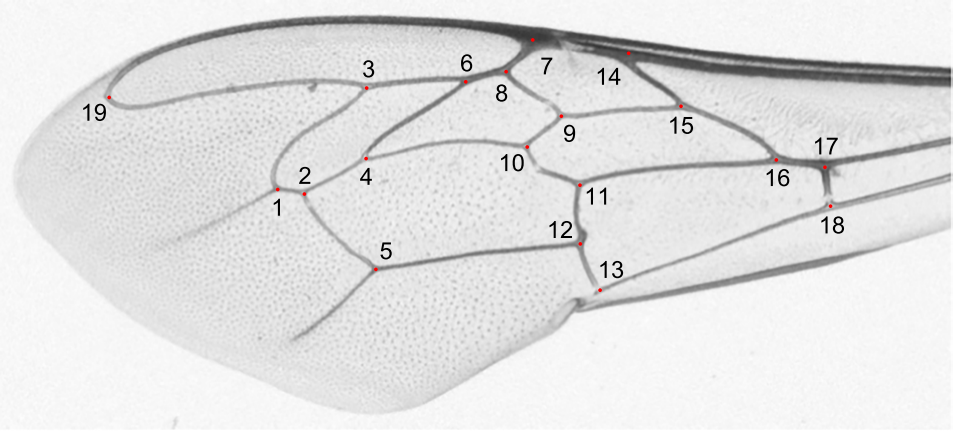
Forewing of a honey bee worker showing the position of landmarks marked by numbered red dots.

After collection, the workers were stored in alcohol until preparation. The alcohol concentration and wing hydration at the time of measurement differed between earlier studies, which could affect wing shape measurements to some degree. The forewings were detached from the body and mounted on microscope slides. Wing images were obtained using different equipment and saved in different resolutions. Before landmark determination, they were saved in PNG format. The wing images were analyzed using the IdentiFly software^37^, which is freely available at http://drawwing.org/identifly. On each wing 19 landmarks were determined (Fig. 2). The landmark’s configuration is the same as in earlier studies^37,38^. The wings used in this study were measured by the same person (JM). To estimate measurement error, the landmark coordinates were automatically adjusted three times using template matching^39^. Three wing images from three different colonies were used as templates. The estimation of measurement error was based on a subset of wing images from Georgia. The subset contained 1,049 wings representing thirty colonies. Each colony was represented by at least thirty workers.

### Statistical analysis

Statistical analysis was performed using the R statistical computing environment (version 4.5.0)^40^ with the RStudio integrated development environment (version 2025.05.0). The landmarks were subjected to geometric morphometric analysis. The raw landmark coordinates were aligned using the generalized Procrustes method as implemented in the geomorph package (v. 4.0.10)^41^. Due to the relatively large variation between workers (see below for details), the aligned coordinates were averaged across colonies. These averages were then subjected to statistical analysis. Principal component analysis (PCA) was performed to determine the first 34 principal components used to describe wing shape. Differences between countries were analyzed by multivariate analysis of variance using the manova function from the stats package and linear discriminant analysis using the Morpho package (v. 2.12)^42^. Measurement error was estimated using the gm.measurement.error function^43^ from the geomorph package (v. 4.0.10)^41^. The error analysis was based on the aforementioned subset of thirty colonies from Georgia. The same subset was used to estimate differences in wing shape between workers and colonies. Wing shape differences were quantified using Procrustes distance, calculated as the square root of the sum of squared deviations between corresponding landmark positions after Procrustes alignment. For each colony, the average of the pairwise differences between all workers was calculated. The mean of these thirty averages was used as an estimate of the Procrustes distance between workers. The mean of the pairwise differences between the thirty colonies was used as an estimate of differences between colonies. The Procrustes distance between countries was calculated using the whole dataset as the mean value of the pairwise differences between the nine countries. We used one colony from Georgia (GE-0001) to verify how the number of workers we sampled from a colony affects classification. From the colony, 36 samples were taken. These consisted of one, two, four, eight, 16 or 32 workers. The samples were taken without replacement. All colonies were classified as evolutionary lineages: A, C, M and O, by comparing them with reference samples obtained from the Morphometric Bee Database in Oberursel^37^ using the IdentiFlyR package (v. 0.1.1)^44^. The reference samples for the lineage consisted of 1,849 workers from 187 colonies. Full details of the statistical analysis are available as an R Markdown document^24^.

## Data Records

We provide here the complete set of wing images and their associated data. For each country, there are three files beginning with the two-letter ISO 3166-1 country code: ‘XX-wing-images.zip’, ‘XX-raw-coordinates.csv’ and ‘XX-data.csv’. The first of these contains wing images, the second contains raw landmark coordinates and the third contains geographic coordinates. The wing images have been sorted by country and compressed into ZIP files. Each wing has been saved as a separate image file with the.DW.PNG extension. When opened with IdentiFly software, both wings and landmarks are visible. The images can also be opened in any image viewer or editor that is compatible with the PNG format; however, in this case, the landmarks may remain invisible. The filenames of the wing images begin with the two-letter country code, followed by a hyphen, a four-digit sample code, another hyphen and finally the original filename. As the samples were assigned an independent number in each country, the unique sample name must include the two-letter country code.

Each line of the CSV files corresponds to one wing, and the data fields within each line are separated by commas. CSV files can be opened in a variety of spreadsheet editors and can also be easily imported into R software^40^, as demonstrated in the R markdown document^24^. A CSV file containing raw landmark coordinates consists of 39 columns. The first column (labelled ‘file’) contains the name of the wing image file, and the remaining 38 columns (labelled ‘x1’, ‘x2’, ‘y1’, ‘y2’,…, ‘x19’, ‘y19’) contain the X and Y coordinates of the 19 landmarks. The raw landmark coordinates are positive integers, with units of pixels. They represent the position of a landmark in the Cartesian coordinate system, with the origin at the lower left corner of the image. A CSV file containing data consists of seven columns. The first column (labelled ‘file’) contains text indicating the name of the wing image file. The second column (labelled ‘sample’) contains text indicating the colony. Columns three and four (labelled ‘latitude’ and ‘longitude’) contain the geographic coordinates of the location from which the sample was obtained. These coordinates are in the format of decimal degrees. Latitude can range from −90.0 to 90.0 degrees, and longitude can range from −180.0 to 180.0 degrees. The fifth column (labelled ‘date’) contains the year in which the sample was collected. It is in the form of a four-digit integer. The sixth column (labelled ‘resolution’) contains information about the scale of the image. This indicates how many pixels in the image correspond to one meter in reality. The seventh column (labelled ‘notes’) can contain various textual information. The first five columns always contain information, but the last two columns can be empty. The complete dataset, including wing images, landmark coordinates, geographic coordinates of sampling locations and other data, is available on the Zenodo website^45^.

## Technical Validation

**Fig. 3:**
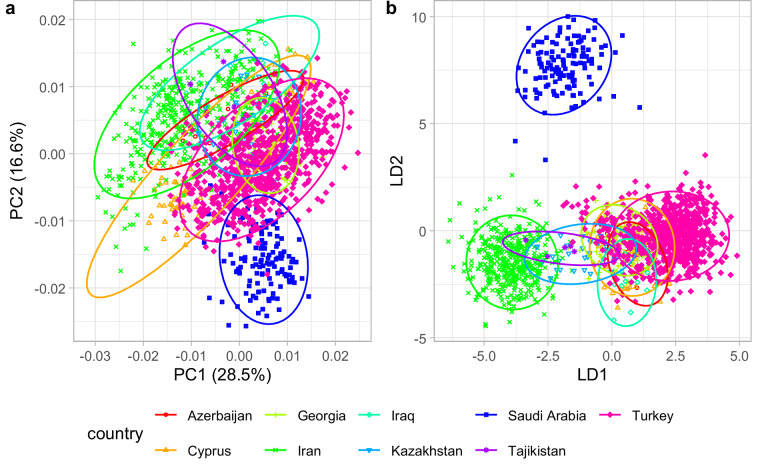
The first two principal components (**a**) and the first two linear discriminants (**b**) of wing shape. Each marker represents a colony. The 95% confidence regions are indicated by the ellipses.

A multivariate analysis of variance revealed significant differences in the wing shape of honey bees from the nine countries (F = 31.3, P < 10^−15^). Pairwise comparisons showed significant differences between most countries (Table 1). The only non-significant difference was between Tajikistan and Kazakhstan (Table 1). The dissimilarities among the nine countries are also evident in the plots of the first two principal components (Fig. 3a) and the first two linear discriminants (Fig. 3b). While the points representing different countries can overlap to some degree in the graphs of the first two dimensions, the wing shape differs between countries in other, invisible dimensions. With all dimensions included, most colonies can be correctly assigned to their respective countries. Using leave-one-out cross-validation, the correct classification rate was 96.7% (see the R Markdown document^24^ for details).

**Table 1:** Differences in wing shape between countries expressed as Procrustes distances (lower triangle) and significance of pairwise comparisons (upper triangle).

Country	AZ	CY	GE	IR	IQ	KZ	SA	TJ	TR
Azerbaijan (AZ)	—	10^−04^	10^−04^	10^−04^	1.5 × 10^−03^	10^−04^	10^−04^	2.0 × 10^−04^	10^−04^
Cyprus (CY)	0.0154	—	10^−04^	10^−04^	10^−04^	10^−04^	10^−04^	10^−04^	10^−04^
Georgia (GE)	0.0195	0.0182	—	10^−04^	10^−04^	10^−04^	10^−04^	3.1 × 10^−03^	10^−04^
Iran (IR)	0.0161	0.0133	0.0205	—	10^−04^	10^−04^	10^−04^	4.4 × 10^−03^	10^−04^
Iraq (IQ)	0.0108	0.0170	0.0189	0.0164	—	10^−04^	10^−04^	2.0 × 10^−04^	10^−04^
Kazakhstan (KZ)	0.0174	0.0173	0.0117	0.0151	0.0157	—	10^−04^	1.1 × 10^−01^	10^−04^
Saudi Arabia (SA)	0.0291	0.0235	0.0229	0.0277	0.0321	0.0236	—	10^−04^	10^−04^
Tajikistan (TJ)	0.0149	0.0180	0.0155	0.0128	0.0128	0.0095	0.0284	—	5.0 × 10^−04^
Turkey (TR)	0.0145	0.0132	0.0118	0.0185	0.0162	0.0127	0.0215	0.0163	—

**Fig. 4:**
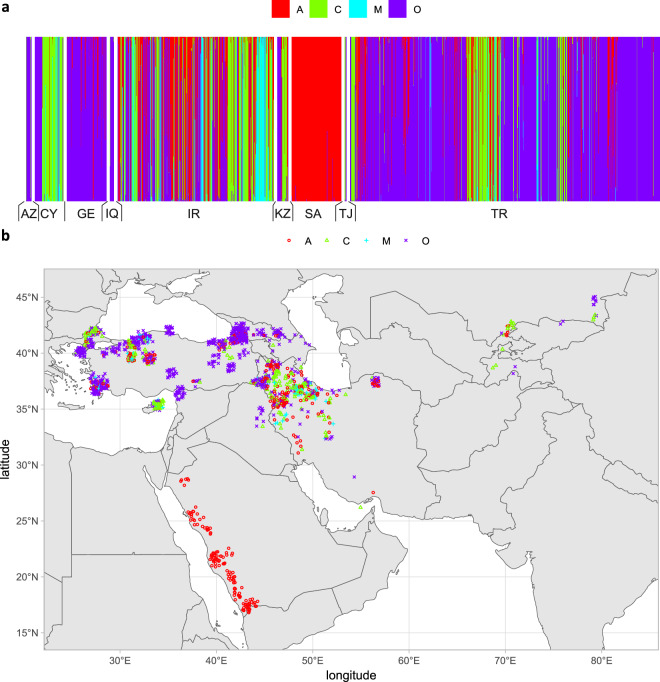
Colonies were classified into four evolutionary lineages, as shown in the admixture graph (**a**) and map (**b**). Abbreviations: AZ - Azerbaijan, CY - Cyprus, GE - Georgia, IQ - Iraq, IR - Iran, KZ - Kazakhstan, SA - Saudi Arabia, TJ - Tajikistan, TR - Turkey.

Using reference samples from the Morphometric Bee Database in Oberursel, the colonies were classified into four evolutionary lineages: A, C, M, and O. Most colonies (53.2%) were classified as lineage O, while many were also classified as lineage A (24.6%), C (16.7%), or M (5.5%) (Fig. 4, Table 2). Lineage O is native to most of the study area, excluding the Arabian Peninsula and the European part of Turkey. It is relatively abundant in Georgia (93.1%), Iraq (90.9%), Azerbaijan (85.7%), and Turkey (73.2%) (Table 2), indicating that conserving honey bee biodiversity within lineage O is possible. Another country with low introgression is Saudi Arabia, where all colonies were classified as lineage A, which is native to the region. We also found many signs of introgression. Lineage C was present not only in the European part of Turkey, where it can be considered native, but also in other regions. A particularly large proportion of colonies classified as lineage C was found in more recent samples from Cyprus. A relatively large proportion of M lineage colonies were found in Iran, despite this lineage not being native to the study area. This is surprising, as most beekeepers nowadays avoid lineage M and prefer lineage C and its hybrids. The presence of lineage M in Iran may be related to historic British influence in the country.

**Table 2:** The number of colonies from nine countries in southwestern Asia was classified into four evolutionary lineages.

country	lineage			
	A	C	M	O
Azerbaijan	0	2	0	12
Cyprus	2	43	8	20
Georgia	7	0	0	95
Iraq	0	1	0	10
Iran	146	107	52	96
Kazakhstan	6	11	0	12
Saudi Arabia	127	0	0	0
Tajikistan	0	3	0	2
Turkey	95	93	25	582

**Fig. 5:**
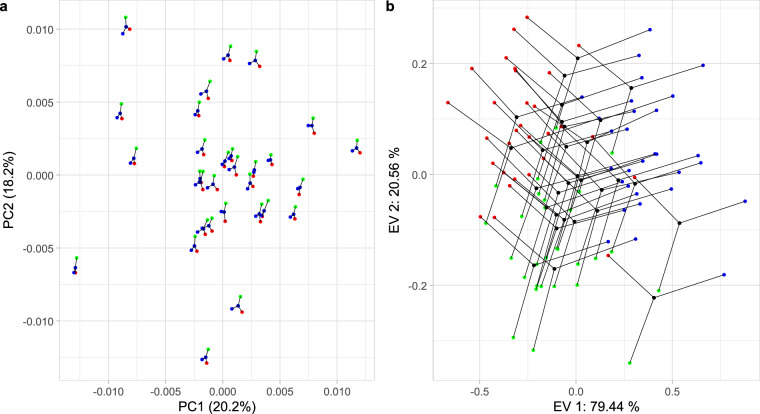
Three replicated measurements of thirty colonies are presented in the graph of the first two principal components (**a**) and the first two signal-to-noise ratio eigenvectors (**b**). Red, green, and blue dots represent three replicate measurements of a colony, while black dots represent the colony mean. Dots representing the same colony are connected by black lines.

The analysis of measurement error was based on three replicate measurements from thirty colonies. Results indicated a significant and relatively large systematic error (Table 3), whereas random error was much smaller (Table 3). The graph of the first two principal components shows that the replicated measurements of a single colony form clusters, and the differences between colonies are clearly visible (Fig. 5a). However, if the same data are presented in a graph of the first two signal-to-noise ratio eigenvectors, the differences between replications are larger than the differences between colonies (Fig. 5b). The relatively large systematic error can lead to different results based on measurements obtained by different operators. In this situation, it is recommended that the landmarks be remeasured by the same person or computer algorithm when combining the data with other data. Wing images provided in this study allow their remeasurement either manually or automatically. In this way measurement error related to operator can be markedly reduced.

**Table 3:** Analysis of variance table provides an evaluation of the random and systematic components of measurement error.

	Df	R^2^	ME^2^	SNR	Z	P
Colonies	29	0.919		266.3	17.9	0.001
Systematic ME	2	0.077	0.957	22.4	13.8	0.001
Random ME	58	0.003	0.043			
Total	89					

Abbreviations: ME – measurement error, SNR - signal-to-noise ratio.

In addition to operator error, device-related error may arise from the use of different types of equipment to capture the wing images. The wing images provided here were obtained using various equipment, so the source of the error remains unknown. Earlier studies showed that device-related measurement error was much smaller than operator-related measurement error^46,47^. Another factor that can affect measurement error is the method of wing preparation, including the hydration of wings prior to measurement. Wings intended for measurement should be flattened between two microscope slides. Flattening is easier if the wings are hydrated and flexible.

The mean Procrustes distance between individual workers (0.0231) exceeded those between colonies (0.0141) and countries (0.0177). In contrast, the Procrustes distance between replications used to estimate measurement error was much smaller at 0.0053. Relatively large interindividual variation can complicate the classification of honey bees at the levels of geographical region, subspecies, or evolutionary lineage. To reduce this variation, a larger number of workers should be obtained from a colony, and their averages should be used for classification.

**Fig. 6:**
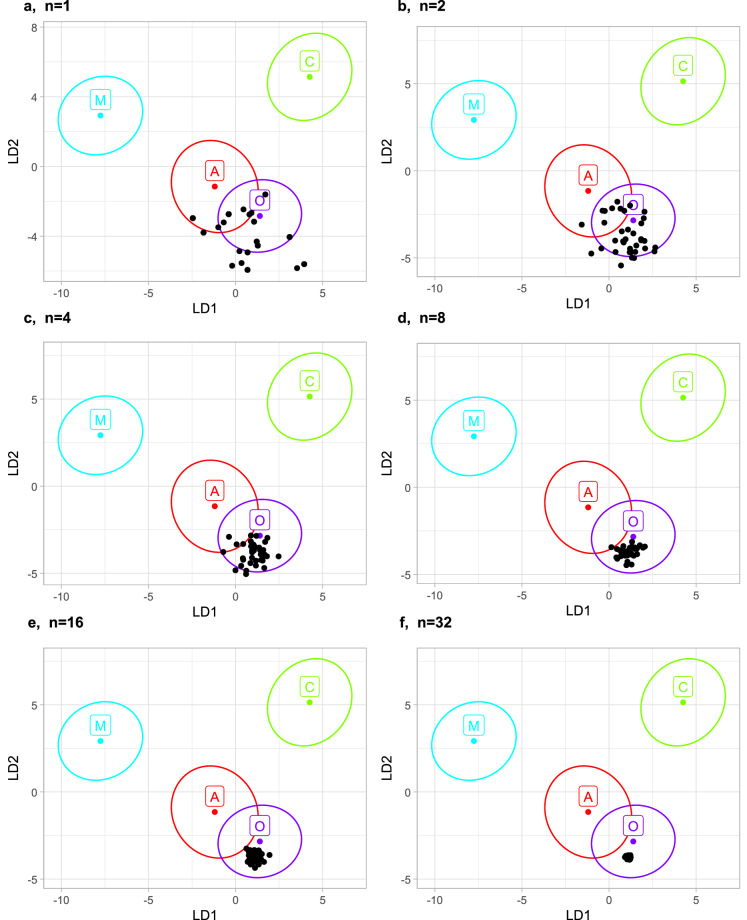
Classification of samples as evolutionary lineages is illustrated by the first two linear discriminants. The samples were created by resampling without replacement. They originated from one colony and consisted of one (**a**), two (**b**), four (**c**), eight (**d**), 16 (**e**), and 32 (**f**) workers. As the sample size increases, the variation of the points decreases. Most samples were classified as lineage O, while only two samples consisting of a single worker and two samples consisting of two workers were classified as lineage A.

One colony from Georgia was used as an example of classification into evolutionary lineages. When classification was based on single workers from the colony (Fig. 6a), the results varied markedly. As the sample size increased, the variation in results decreased (Fig. 6b–f). This also demonstrates that classification into lineages can be reliable despite the significant variation between workers; however, it must be based on the averages of a large number of workers from one colony.

## Usage Notes

The data provided here can be used in studies of honey bee biogeography, with special emphasis on protecting native subspecies and ecotypes from extinction. The landmark coordinates can be used directly in comparative studies if the same configuration of landmarks is used in future studies. However, to avoid systematic measurement error, it is recommended that the landmarks be remeasured by the same person or computer algorithm. The R Markdown document^24^ with statistical analysis illustrates the use of the landmark data.

Although the dataset is insufficient to reconstruct pre-introgression populations, the current wing morphology retains a meaningful geographic signal, allowing for the creation of a valuable, practical monitoring tool. The wing images provided in this study can effectively verify whether a focal colony originated from a particular country or was recently introduced. This verification can be used to enforce a ban on imports, which is important for biodiversity conservation. To conduct this verification, a sample of at least ten workers should be collected from a verified colony, and their wing images should be obtained. Landmarks should then be determined on the wing images and compared with the data provided in this study. The comparison is performed using IdentiFly software and identification files, which are easily created in R with the IdentiFlyR package^44^. Result of this procedure is classification of a colony either as local or recently introduced. This method will be effective in most cases because honey bees differ markedly between countries (Table 1), and a large proportion of the colonies were correctly classified by cross-validation as belonging to the appropriate country.

A similar methodology can be used to eliminate introgressed colonies from the local population. This task is more difficult than detecting imported colonies because it is usually unclear what the phenotype of native bees was. For example, in Iran, only 23.9% of the colonies were classified as the native O lineage. These colonies likely differ from the original population, but it can be assumed that they resemble their ancestors. Selecting which colonies should be included in the reference population of native bees should involve genetic markers. Once the reference population is established, wing morphometry can be used to classify colonies as native or non-native. Detecting native honey bees is associated with greater uncertainty, but it can be effective in practice when only a small fraction of clearly non-native colonies are requeened each season.

In the future, standardizing the wing image acquisition and landmark detection processes should minimize measurement error. Operator error can be reduced by applying recently developed, artificial intelligence-based automated landmark detection^48,49^. The large dataset of wing images presented here should facilitate the development of even more effective methods. It is important to accompany the newly obtained wing images with molecular markers. Integrating morphometric data with molecular markers will provide deeper insights into pre-introgression populations.

## Data Availability

The complete dataset, including wing images, landmark coordinates, geographic coordinates of sampling locations and other data, is available on the Zenodo website^45^.

## Code availability

A GitHub repository containing an R Markdown document^24^ with details of the statistical analysis is available at https://github.com/DrawWing/Apis-mellifera-Asia. Most of the figures and tables in this publication were generated using this code.
